# Clinical research with probiotics as an indicator of global valorization since the year 2000

**DOI:** 10.3389/fmicb.2023.1323920

**Published:** 2023-12-14

**Authors:** Cato Wiegers, Eveline H. T. van Beek, Olaf F. A. Larsen

**Affiliations:** Athena Institute, Vrije Universiteit Amsterdam, Amsterdam, Netherlands

**Keywords:** clinical trials, probiotics, gut microbiota, market, valorization

## Abstract

Probiotics are becoming increasingly popular due to their potential health benefits. With this rise in popularity and demand as indicated by ever-growing market prospects, it seems evident that innovation and valorization are on the rise as well. However, an increasing body of literature shows that innovation is stagnating, which may be detrimental to the exploitation of the benefits of probiotics, for example the development of alternative therapies to manage the increasing prevalence of metabolic and autoimmune disorders. To this end, this study investigated global clinical trials that have been executed since the year 2000 as a first indicator of the status of probiotic valorization. The cumulative number of clinical trials has indeed increased significantly from 0 at the start of the century up to 2,517 registered trials in 2023. However, in Asia, Europe, and North America, the continents with the highest numbers of clinical trials, stagnating or declining trends have been found. In these locations, most clinical trials were funded by non-industry sponsors and targeting probiotic supplements or undefined products. Considering the overall stagnation in clinical trials and viewing these trends in the context of developments in local markets and regulations, the global valorization of probiotics appears to slow down. This could impact the transition from academic research to the development of products that are beneficial and accessible for consumers, either to maintain a healthy lifestyle or to treat medical conditions.

## 1 Introduction

Since the start of this century, probiotics, defined as “live microorganisms that when administered in adequate amounts confer a health benefit on the host” (Sanders, 2008; Hill et al., 2014), have seen a rise in popularity (Sanap et al., 2019). This is reflected by a steadily growing global market, which is estimated to grow even further in the coming years. In fact, in 2021 the global probiotic market was estimated to be worth US$47.6 billion, and in 2022 the European market had an estimated value of €9.4 billion, demonstrating a growth of over 9% compared to 2018 (International Probiotics Association Europe, 2023).

The growing interest in probiotics as reflected by the market size may be due to their many possible applications as well as their safety (van den Nieuwboer et al., 2014, 2015a,b; Sanap et al., 2019). Evidence suggests that probiotics show potential as a treatment or prophylaxis for a multitude of illnesses and disorders. These include hospital acquired infections, cancer, pancreatitis, necrotizing enterocolitis, *Helicobacter pylori* infections, irritable bowel syndrome, autism spectrum disorder, and numerous other infectious and autoimmune diseases (McFarland, 2015; Liu et al., 2018; Chong et al., 2019; Ansari et al., 2020; Tegegne and Kebede, 2022). According to the International Scientific Association for Probiotics and Prebiotics (ISAPP), mechanisms of action of probiotics include colonization resistance, production of acid and short-chain fatty acid, regulation of intestinal transit, normalization of perturbed microbiota, increased turnover of intestinal epithelial cells, and competitive exclusion of pathogens (Hill et al., 2014).

Despite the high potential of probiotics, research suggests that the valorization and industrial development of probiotics is hampered (van den Nieuwboer et al., 2016; International Probiotics Association Europe, 2018). In this case, valorization is defined as “the process of creating value from knowledge, by making knowledge suitable and available for societal and/or economic application and by transforming it into competitive products, services, processes and new business.” (Vrije Universiteit Amsterdam, 2023). The slowing pace of valorization of probiotics may be due to a number of research related causes, such as the lack of guidelines and standards in research, or the high inter- and intrapersonal variation regarding the effects of probiotic products. Other reasons why the probiotic industry may be halted in its progress have been given by van den Nieuwboer et al. (2016), including a lack of knowledge on mechanisms of action, a lack of biomarkers, poor collaboration between stakeholders, logistical difficulties, and regulatory factors.

It has been suggested that European regulations as executed by the European Food Safety Authority (EFSA) may have a negative impact on the development of the field of probiotics. For example, a significant decrease in probiotic sales was demonstrated in Europe after the implementation of the Nutrition and Health Claims Regulation in 2006 (Regulation (EC) No 1924/2006), while countries outside of Europe demonstrated a continued growth in sales (International Probiotics Association Europe, 2023). Moreover, another important barrier related to EFSA regulations is the fact that to be able to make a health claim about a probiotic product, a health claim dossier needs to be submitted to the European Commission. However, requirements about this dossier are unclear, and since 2009, all but one of these dossiers have been rejected by the EFSA (Janmohamed, 2023). Additionally, the approved health claim does not concern a product that is sold as a probiotic, but yoghurt and its effects on lactose digestion (Food Supplements Europe, 2021).

However, despite the suggested hampering of the industry, the global market is estimated to continue growing steadily at a rate of 8.9%, reaching a worth of US$73.14 billion in 2023 (Research and Markets, 2023). To investigate how this (lack of) growth is developing and to gain insight into the status of valorization of probiotics, this study will provide an overview and in-depth analysis of global clinical trials since the year 2000. Clinical trials can be regarded as a measure of valorization, as they reflect late-stage research and are often a final step in the research process before a product enters the market (Ramezanpour et al., 2015; Janse et al., 2020). Therefore, by studying clinical trials, it is possible to provide an estimate of the global valorization of probiotics.

## 2 Materials and methods

With the aim of providing an overview of all clinical trials that have been executed with probiotics, two commonly used clinical trial databases were utilized. Firstly, the World Health Organization (WHO) International Clinical Trials Registry Platform (ICTRP) consists of a number of different clinical trial registries that fit the WHO criteria, ensuring a large amount of high-quality clinical trial data (World Health Organization, 2023.). Secondly, the U.S. National Library of Medicine (NLM) ClinicalTrials.gov database contains quality controlled clinical trial data from over 200 countries, with elaborate search options (National Library of Medicine, 2023).

### 2.1 Clinical trial data collection

The selected databases were consulted to search for clinical trials that have been conducted since the year 2000. This timeframe was chosen based on a crude search in the databases, indicating that the number of clinical trials that have been executed before the year 2000 is negligible. A general search query was formulated to include the most popular probiotic organisms (National Institutes of Health, 2022): *(“Probiotic” OR “Probiotics”) OR (“Lactobacillus” OR “Lactic acid bacterium” OR “LAB”) OR “Streptococcus” OR “Bifidobacterium” OR “Enterococcus” OR (“Escherichia” OR “E. coli”) OR “Saccharomyces.”*

To ensure all possible clinical trials related to probiotics were included, OR operators were used as much as possible, and advanced search options were used. The date of registration was set at January 01, 2000, and the search string was only applied to the intervention field, leaving the rest of the search blank. After downloading the results, all records were scanned for duplicates. Additionally, all records were checked for suitability based on the inclusion requirement of stating the use of any form of probiotic product as an intervention, regardless of the studied condition.

### 2.2 Clinical trial data analysis

Clinical trial records were investigated and manually coded based on the type of sponsor, start year, location, and type of probiotic that was used. In this study, the type of sponsor refers to the funder of the clinical trial. The sponsor types were defined as three categories consisting of (1) industry, such as commercial companies, (2) non-industry, such as academia and other governmental organizations and (3) public-private partnerships (PPP), consisting of combinations of industry and non-industry sponsors. The start year of the clinical trials was analyzed by checking the indicated date of either enrollment or trial start, as this was considered to provide more accurate information than the date of registration. Trial location was defined as the country or countries where the clinical trial took place, which was stated either directly or traceable through any provided contact information.

Finally, the type of probiotic was divided in 4 categories, including (1) food, (2), non-food, (3) supplement, and (4) undefined. The food category was used for probiotic foodstuff such as dairy products and beverages. Non-food includes clinical trials using probiotic drugs or probiotics that are not meant to be ingested orally, such as suppositories and topical creams. This category also includes clinical trials using live biotherapeutics, defined as products containing live microorganisms to treat or prevent disease (Cordaillat-Simmons et al., 2020). Supplements include probiotics that can be bought without a prescription, meant for oral ingestion without any additional nutritional values that a probiotic food might have. Lastly, the undefined category was used to include clinical trials that did not specify a probiotic strain or product type, but for example simply stated the word probiotics.

Each of the indicated characteristics were inventoried for each year to be able to visualize trends over time. Since the data collection did not include the full calendar year of 2023, the decision was made to include all clinical trials executed from January 01, 2000 to December 31, 2022. Additionally, the choice was made to group clinical trial locations per continent, leading to a division between Africa, Asia, Europe, North America, South America, and Oceania. To reduce possible noise and indicate general trends, moving averages were calculated to generate trendlines based on the average of 2 subsequent data points. Since the bulk of clinical trials were conducted in only 3 continents, the choice was made to exclude Africa, Oceania, and South America from the data visualization regarding sponsor and product types.

To further investigate possible drivers for the valorization of probiotics, a sub analysis was performed on the clinical trials that were registered in Europe. Clinical trials from countries that are part of the European Union (EU) were filtered by sponsor- and product type, focusing on industrial sponsors. The EU was chosen as a focal point since the number of clinical trials from Europe was deemed large enough and the EU has a single dedicated regulatory body that handles probiotics (EFSA).

## 3 Results

**Figure 1:**
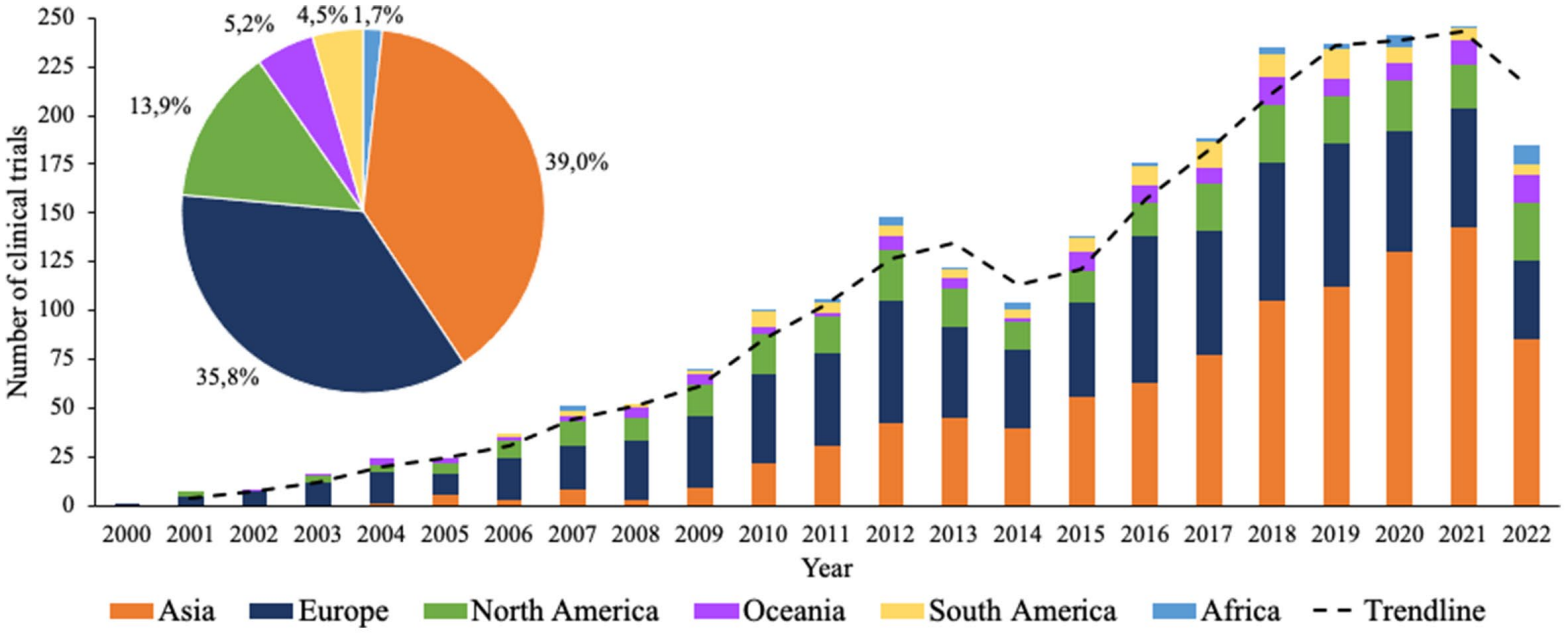
Timeline of all clinical trials with probiotics registered since 2000. Bars indicate the absolute number of clinical trials for each continent. Pie chart indicates the total percentual contribution of each corresponding continent.

In total, the two databases yielded 5,359 clinical trial records, of which 2,517 clinical trial records were included for analysis. The main reasons for exclusion of clinical trials were stating conventional drugs as the sole intervention, stating probiotics usage as an exclusion factor, or duplicate results. An overview was made of the number of clinical trials that were initiated each year in each continent. Figure 1 shows that the overall largest numbers of clinical trials were executed in Asia (39.0%), Europe (35.8%), and North America (13.9%). Considering the development over time, Europe appears to be at the forefront, with the highest number of clinical trials in 2001 until approximately 2013, after which Asia starts to show rapid growth while clinical trials in Europe and North America seem to stabilize. Additionally, clinical trials have been executed to a lesser extent but at a steady rate in Oceania since 2003, in South America since 2006, and in Africa since 2009. Interestingly, for all continents it appears that the rate of growth established from 2001 collapsed between 2012 and 2014, after which it started to rise again.

**Figure 2:**
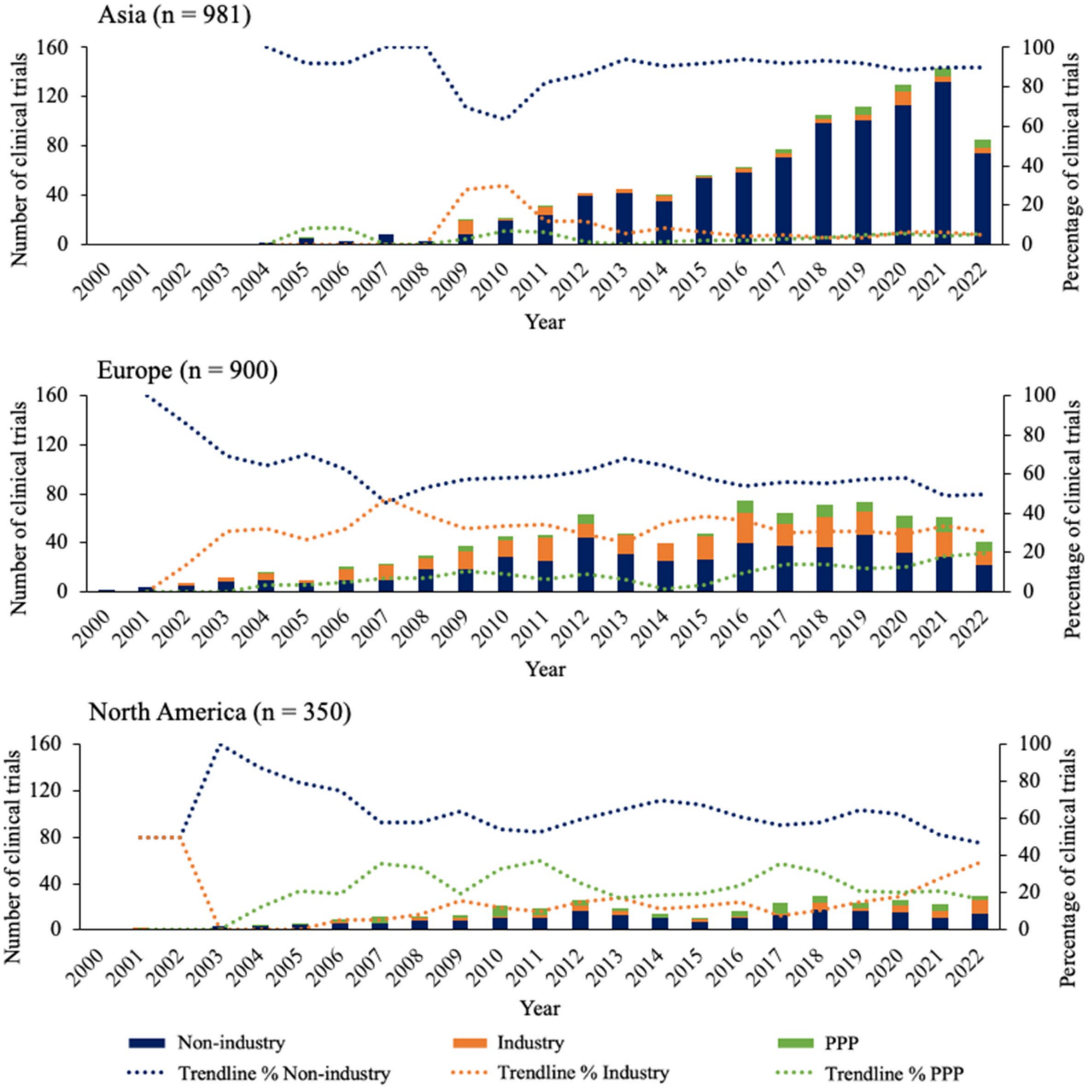
Overview of sponsor types for clinical trials in top 3 continents since 2000. Bars indicate the absolute number of clinical trials for each sponsor type. Trendlines are based on average percentage of two subsequent years.

Focusing on the sponsor types of the clinical trials executed in Asia, Europe, and North America, Figure 2 shows distinct patterns for all 3 continents over time. Starting with Asia, the majority of clinical trials have been sponsored by non-industrial parties from 2004 onwards, plateauing to a percentual contribution of around 90% in 2014. The percentage of industrial sponsors shows to increase from 2008, but then stabilizes around 2014 at ~5% together with public-private partnership (PPP) sponsors. In Europe, non-industry sponsors also make up the majority, however to a lesser extent compared to Asia. In fact, the contribution of non-industry sponsors declines from 2001 until it reaches the level of industry sponsors at approximately 45% in 2007. After this, a slight growth can be seen for non-industry and PPP, while industry sponsors decline in number. In North America, non-industry sponsors follow a similarly stable pattern over time compared to Europe, however North America is the only location where the contribution of PPP sponsors is relatively larger than that of industry sponsors for most years, until 2021 where it intersects with industry sponsors at approximately 20%.

**Figure 3:**
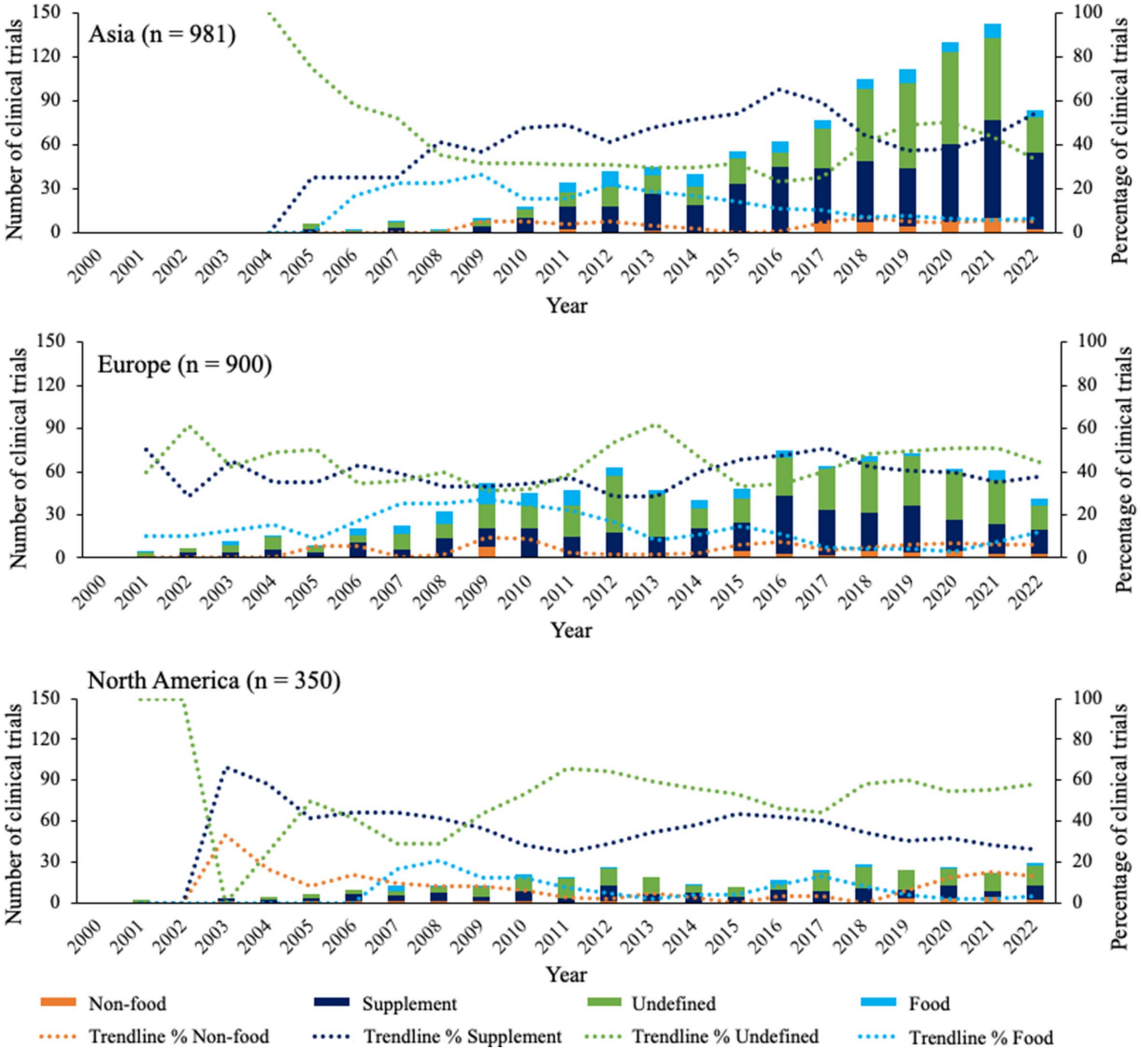
Overview of product types for clinical trials in top 3 continents since 2000. Bars indicate the absolute number of clinical trials for each product type. Trendlines are based on average percentage of two subsequent years.

Figure 3 shows the trends of different probiotic product types in Asia, Europe, and North America. Overall, the three continents showed similar trends for all product types, with the majority of clinical trials focusing on supplements or undefined probiotics, the latter making up ~35% to ~60% of all clinical trials. In North America and Europe, it seemed that clinical trials with undefined products increased around 2011 and again around 2017. In Asia, this trend was less visible, however for supplements a similar decline was seen from ~2016 for all 3 continents. Additionally, percentages of clinical trials with probiotic food and non-food remained below 30%, showing a decline or stabilization from approximately 2009 in Asia, Europe, and North America.

**Figure 4:**
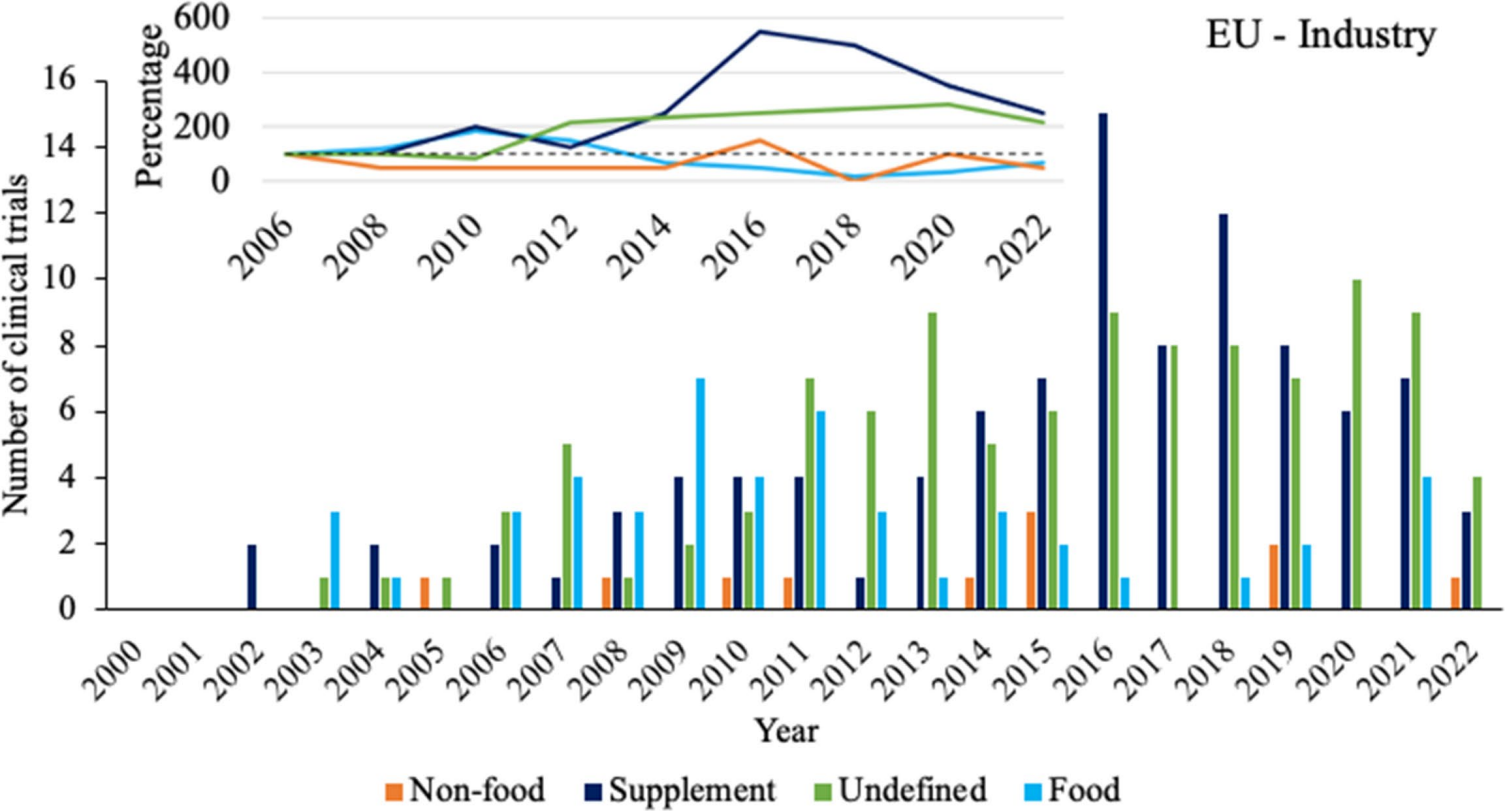
Overview of product types for clinical trials with sponsors from the industry in the European Union. Bars indicate the absolute number of clinical trials for each product type. Insert shows trendlines based on calculations of percentual changes compared to 2006, taking the average number of clinical trials from two subsequent years.

Finally, a sub-analysis was performed for clinical trials funded by industrial sponsors and executed in the EU to serve as a case study with a relatively high number of clinical trials (*n* = 263) under a single regulatory body (EFSA). The year 2006 was taken as the starting point for analysis, as this is the first year that clinical trials with at least 3 out of 4 product types have been registered and all product types have been registered at least once. Figure 4 shows that compared to 2006, clinical trials with supplements and undefined probiotic products have gradually doubled in 2022. Contrastingly, clinical trials with food products seemed to increase until approximately 2009, after which the number of clinical trials started to plateau and decrease. Clinical trials with non-food probiotics have always been the lowest in number, fluctuating between 0 and 3 per year.

## 4 Discussion

The aim of this study was to provide insight into the status of valorization of probiotics by investigating clinical trials that have been executed since 2000. It was found that the largest contributors of clinical trials with probiotics were Asia, Europe, and North America, showing considerable growth over the past 20 years. For all 3 of these continents, most clinical trials were funded by non-industry sponsors, followed by industry and public-private partnerships, though in different proportions. The most popular interventions were supplements and undefined probiotic products, followed by probiotic food and non-food products.

### 4.1 Global trends in clinical research with probiotics

Overall, the findings of this study are in accordance with the trends presented in the study by Dronkers et al. (2020), indicating Europe, North America, and Asia as the locations where the highest numbers of clinical trials are executed. Furthermore, these findings align with market research data, highlighting the high increase in interest in probiotics over the past decades. Some of the reasons for this may be the increasing awareness of the health benefits of probiotics, as well as a growing interest in taking a more “proactive” approach to health, using food as a means to prevent lifestyle related metabolic diseases such as type-2 diabetes and obesity (Vodnar et al., 2019; Yamashima et al., 2020; Grand View Research, 2023).

One might assume that in order to meet this growing demand, the industry, as a proxy for the development and marketing of new products, would grow as well. However, when it comes to the different financial sponsor types that were indicated in the studied clinical trial records, it was found that the highest number of clinical trials were funded by governmental organizations (non-industry). Remarkably, while in Europe and North America the contribution of non-industry sponsors appeared to fluctuate between 50 and 60%, in Asia the contribution reached up to and over 90%. The reason for this large difference is not evident, but it might be related the registration process of clinical trials into the utilized databases. For example, it may be possible that some of the clinical trials that are indicated as funded by non-industrial sponsors may actually be (partially) funded by industrial sponsors. A possible consideration for doing so could be that it is sometimes presumed that clinical trials funded by industry and commercial parties may be at risk of bias towards more positive results (Barden et al., 2006).

Focusing on the past 5 years, it appears that globally, the number of clinical trials stabilized from 2018 onwards. A possible contributing factor could be saturation of the intellectual space. This may be the result of increasing convergence between companies that has been observed in probiotic innovation, indicating that knowledge is being shared between different industries (Bornkessel et al., 2014). Another possible cause of the lack of growth of the number of clinical trials in recent years could be the high costs related to clinical research. Before a product is introduced to the market, time and funds are required for proof-of-concept, quality, safety and legislative evaluation, and upscaling (van den Nieuwboer et al., 2016). Especially for industrial parties, the consideration of funding a clinical trial is closely related to the sales prospects of the product that is to be studied, and with the high number of probiotics on the market this also requires competitive pricing (Kohut et al., 2021).

Regarding the valorization of probiotics, the industry plays a key role in the creation of new business and operationalizing academic knowledge into a process or product that is beneficial for society (van de Burgwal et al., 2018). Therefore, focusing on clinical trials funded by industrial sponsors may be an indicator of the rate of valorization of probiotics. This was further investigated by studying clinical trials in the EU, due to having the overall largest number of clinical trials funded by the industry (*n* = 263).

### 4.2 The possible impact of regulations

Specifically in the EU, an important factor that may contribute to the identified stabilizing or declining trends could be the regulations that probiotic foods and supplements are subjected to (European Food Safety Authority, 2023a,b). The regulatory approach taken on by the EFSA has been deemed a barrier for the valorization of probiotics, and several articles have addressed the need for strategies to adhere to the regulations (Gibson et al., 2011; Binnendijk and Rijkers, 2013; Kumar et al., 2015; van den Nieuwboer et al., 2016). Particularly, since the introduction of the regulation on nutrition and health claims made on foods in 2006 (European Parliament and the Council of the European Union, 2006), there has only been one health claim approval for probiotics (more specifically, yoghurt cultures for lactose digestion; Food Supplements Europe, 2021), meaning hundreds have been rejected (International Probiotics Association Europe, 2021).

Interestingly, the timing of this regulation appears to be similar to that of the decline in clinical trials with probiotic foods in the EU. While the regulation was introduced in 2006, the first health claim applications were sent back (either rejected or requiring revisions) to the applicants in 2009 (Janmohamed, 2023), which is the same year after which the number of food related clinical trials starts to decline. This decline in clinical trials may be the consequence of the public nature of these events, making the industry more hesitant to fund new clinical trials during this time period due to poor (financial) prospects associated with the extremely low chances of acquiring approval of a health claim (Forssten et al., 2020). This is also supported by the report on probiotics from Food Supplements Europe (2021), which shows a decline in the proportion of global sales of probiotic supplements in both Western and Eastern Europe between 2013 and 2023.

Moreover, the term probiotics in itself has been deemed a health claim which has not been approved, thus prohibiting use of the word “probiotics” on a product (Quigley, 2022). While the European Commission states that these regulations have been put in place to protect the consumer, International Probiotics Association Europe (2021) states that this lack of communication is making it more difficult for consumers to make responsible choices. Since then, European countries have started to implement national guidelines, adding another layer of complexity to the impact of regulation on the valorization of probiotics (International Probiotics Association Europe, 2023).

### 4.3 Comparing Europe and North America

Remarkably, while in Europe the total number of clinical trials was much higher (*n* = 900) than in North America (*n* = 350), the market prospects of the two continents are very similar. In 2021, the North American market was valued at $11 billion compared to the European market of €9 billion, which is approximately US$10.6 billion using the average currency exchange rate in 2021 (Kamboj and Borah, 2022; European Central Bank, 2023; International Probiotics Association Europe, 2023). It was found that in North America, the percentual contribution of sponsors in the category public-private partnerships (PPP) was higher compared to Europe and Asia. According to van de Burgwal et al. (2018), collaborations such as these are crucial for the valorization of probiotics, and in turn the stimulation of PPP collaborations may lead to the developments of new products. Additionally, considering product types, according to the National Center for Complementary and Integrative Health (2019), probiotic dietary supplements do not require approval by the U.S. Food and Drug Administration (FDA) before marketing, and claims regarding the “structure and function of the body” also do not require FDA approval.

### 4.4 Limitations in clinical research with probiotics

Globally, in addition to supplements, there was a substantial number of clinical trials where the product type was not clearly defined (*n* = 1,043). Information regarding specific strains, dosages and product characteristics were often missing, which has been previously identified as a barrier in probiotics research (van den Nieuwboer et al., 2016; van de Burgwal et al., 2018). This lack of specification of probiotic interventions in clinical research is problematic, as it contributes negatively to the persistent difficulties with showing efficacy of probiotics in clinical research (van den Nieuwboer et al., 2016).

Another issue in this regard is the high inter- and intrapersonal variation between participants in clinical trials with probiotics (Larsen et al., 2020). This high variation could be reduced by implementing changes in the study design, such as ensuring the maintenance of a steady lifestyle (Larsen et al., 2020). However, in general, no detailed information on both intra- and interindividual variation is provided, hampering a more adequate comparison of clinical trials based on stratification of these parameters. Generally, details regarding study design are often left unspecified in clinical trial records, limiting the information to mode of allocation (randomized) and the intervention model (cross-over, parallel, or other). Considering the design of clinical trials with probiotics, the lack of biomarkers has been indicated as another problem (Rijkers et al., 2011). This was confirmed by a high number of outcome variables identified in this study, ranging from self-reported sleep quality to specific inflammatory cytokine levels.

Besides the high variety in different probiotic products and outcome measures, this study included as many clinical trials with probiotics as possible, spanning a wide range of applications from therapies for specific diseases to prevention of illness in healthy individuals. Gaining insight into the optimal combination of these different factors could be beneficial to further facilitate valorization. For example, a review by Homayoni-Rad et al. (2016) indicated that when it comes to clinical research in healthy individuals, probiotic foods may be preferable over supplements when it comes to general health promotion.

### 4.5 Study considerations

Finally, when interpreting the findings of this study, several factors need to be considered. In this case, clinical trials were studied as the main indicator of the valorization of probiotics. While this type of data offers valuable information on the development of the field through late-stage and applied research (Ramezanpour et al., 2015; Janse et al., 2020), there are also other parameters for innovation that have not yet been studied in this context. For example, to the best of our knowledge, there is no data available on the number of products that are available on the market, and on rates of probiotics consumption. Moreover, the transition from knowledge produced in clinical trials into the market entry of products can be considered a prime example of valorization, but it was found extremely difficult to gather information on this. Additionally, the execution of clinical trials may have been disrupted due to recent events such as the COVID-19 pandemic and the war between Ukraine and Russia. While the impact of these events and even more recent conflicts is undisputable, it may still be too early to determine the true effect on probiotic innovation and valorization at this time.

## 5 Conclusion

To summarize, the valorization of probiotics may be stimulated by the growing interest and many possible applications of probiotics. As such, global research and industry have seen significant growth since the start of the 21st century. However, it appears that in recent years this growth has been staggering. Considering the available data on clinical trials, the current field appears to be dominated by academic research on probiotics that are not clearly defined. Confirming the findings of previous studies, this may be due to a lack of innovation, and sustained issues in probiotic research and regulations, suggesting negative drivers for the valorization of probiotics. Given the increase in prevalence of infectious, metabolic, and autoimmune diseases (Larsen et al., 2022), it is important that adequate research and development of possible interventions such as probiotics is stimulated to improve resilience to or to treat these diseases.

## Data availability statement

The original contributions presented in the study are included in the article/supplementary material, further inquiries can be directed to the corresponding author.

## Ethics statement

Ethical approval was not required for the study involving humans in accordance with the local legislation and institutional requirements. Written informed consent to participate in this study was not required from the participants or the participants’ legal guardians/next of kin in accordance with the national legislation and the institutional requirements.

## Author contributions

CW: Formal analysis, Writing – original draft. EB: Formal analysis, Writing – original draft. OL: Conceptualization, Writing – review & editing.
